# Progress of Surgery

**Published:** 1875-02

**Authors:** Jno. E. Owens

**Affiliations:** Lecturer on Surgery, Rush Medical College, Chicago


### Progress in Medical Sciences.

ARTICLE I.

### PROGRESS OF SURGERY.

BY JNO. E. OWENS, M.D.,

LECTURER ON SURGERY, RUSH MEDICAL COLLEGE, CHICAGO.

1. A successful case of Traumatic Tetanus, treated by large doses of Calabar Bean. By SYDNEY RINGER, M.D. (*The Practitioner*, London, November, 1874.)

2. Quinine and Suppuration. By DOUGLAS MORTON, A.M., M.D., Louisville Hospital, Ky. (*The Practitioner*, London, Nov. 1874.)

3. Treatment of Intestinal Obstruction. By Dr. LIBUR. (*Revue Med.-Chir.*, Allemand, 1874; *The Practitioner*, London, Nov. 1874.)

4. Paralysis, caused by Pressure of the India-rubber Cord used in the Bloodless Operation. Reported by Dr. BLAUVELT, House Surgeon at Roosevelt Hospital, N. Y. (*The Med. Record*, Nov. 16, 1874.)

5. Tracheotomy with the Red-Hot Iron. (*Gaz. Med. de Paris; Wien Med. Woch.*, 26, 1874; *The Med. Record*, N. Y., Nov. 16, 1874.)

6. Cases from Prof. GUSTAV SIMON's Clinique at Heidelberg. (Reported for *The Med. Record*, N. Y., Nov. 16, 1874, by NATHAN BOZEMAN.)

1. Dr. O'Leary, Professor of Materia Medica and Therapeutics and of Forensic Medicine at the University of Cork, was attacked with traumatic tetanus, whilst on a visit to London. The case was under the care of Sydney Ringer, M.D., Sir James Paget, and Mr. Gould. When first seen by the former, patient had been ill in bed three days. There was an oval ulcer about two inches long and one and a half broad, the result of a wound received six weeks previously. The ulcer had been irritated a few days before his illness, by putting on riding breeches. During the examination of the leg, several of its muscles became for a few seconds rigidly contracted, and then they relaxed; then in a few seconds the muscles of the opposite thigh became affected, and even the muscles of both legs. Similar attacks occurred every few seconds, affecting sometimes only a few, at other times involving most of the muscles of the legs. The abdominal muscles were much more rarely, and far less severely, affected, and those of the neck occasionally felt stiff. The upper extremities, chest and jaws, were unaffected. The patient protruded his tongue and swallowed readily and without producing tetanic contraction. The spasms were not brought on by external impressions, such as irritating the leg, or by getting out of bed. His pulse was feeble, and beat 120 per minute. His breathing was calm. Thirty grains of chloral, in combination with laudanum, were ordered. The next morning, the fourth day of illness, he was much worse. The spasms, now frequently recurring, involved the arms. The tetanic seizures only remitted, for, on decline of each paroxysm, more or less rigidity still remained. The muscles of the chest and jaw remained unaffected. Extract of physostigma, gr. $\frac{1}{8}$, was now administered every quarter of an hour by the mouth. After five grains had been given, the paroxysms became much less severe, and the attacks intermittent instead of remittent. The expression was much less anxious, the skin moist and slightly perspiring, and the pulse beat 60. During the first twelve hours

(Sept. 13) after having taken 10 grains, the patient was surprisingly better. The medicine had produced slight loss of power, the arms and legs, especially the latter, feeling rather heavy.

Sept. 14. (Second day of treatment.) Last night he took 30 grs. of chloral and 10 drops of laudanum in addition to 1⅓ gr. of ext. calabar bean, every hour, making 20 grains in nineteen hours; patient free from spasms for sixteen hours; no paralysis from the medicine; drug discontinued at 8 P. M.

Sept. 15. At 10 last night paroxysms suddenly returned with terrible severity; calabar bean, ⅓ gr. every quarter of an hour, recommenced; at 10.30 A. M. patient took ⅔ of a grain of extract, which was repeated at 10.40 and at 10.45, after which he took ⅔ gr. every quarter of an hour. The attack continued with great violence, lasting, with slight remissions, from a quarter of an hour to twenty minutes. The patient was in imminent danger of asphyxia. The attacks occurred very frequently, for instance, at 11.00, 11.09, 11.25, 11.45, and 12.25. It being evident that the medicine had not yet produced sufficient effect, at 12.45 P. M. Dr. Ringer ordered every quarter of an hour ℥ss. of a mixture containing ⅓ gr. to ʒi. At 2.45 P. M., symptoms of poisoning becoming manifest, a dose was omitted. Until this time the whole body had been persistently rigid; but now the body is completely relaxed, the jaws being tightly clenched. There is slight general paralysis; he can raise the limbs slowly, but soon they slowly fall again.

At 1 P. M., Sept. 16th, all trismus had disappeared. To this date the seizures had continued with varying frequency and degree, and the dose of the medicine was varied according to its effect, the *object being to keep up just sufficient paralysis to suppress the spasms.*

Sept. 20. The patient was well enough to visit University College Hospital to witness some experiments with the new Brazilian remedy, Jaborandi. He continued to take an occasional dose of his medicine. There is so much that is interesting and valuable to the surgeon in this case that I would fain have reported it at still greater length. The interest is enhanced, on the one hand, by the fact that it was under the care of a most accomplished therapeutist; and, on the other, by the fact that the treatment was attended with success. The spasms first attacked the lower extremities, and gradually ascended the body. It ran an irregular course until the occurrence of the relapse which followed the discontinuance of the medicine, when all irregularity ceased. The attack yielded with unusual rapidity, for in thirty-nine hours after the relapse every vestige of the disease had disappeared. In spite of careful watching, serious general paralysis set in very suddenly, at one time, and that, too, when the patient was not taking the largest doses. In respect to the dose of the bean, Dr. Ringer remarks, this case is less valuable than it ought to have been, on account of a dispensing error. The first bottle contained 10 grains of the alcoholic extract. The *watery* extract, unfortunately, was subsequently used by mistake. In eighty-six hours Dr. O'Leary took 140 grains of the calabar bean; and of this, 88 grains were taken in thirty-two hours—2¾ grains per hour. For a short time he took 4 grains an hour.

Dr. Eben Walton reports a case in which he gave of the alcoholic extract small doses on the first day, but rapidly increased the quantity, so that in a few days his patient took daily 16 grains, then 48 grains, then 57, and on one day 72 grains, without any serious amount of paralysis.

2. Dr. Morton records a series of experiments which he has made with the view of testing the theory that quinine exerts a peculiar influence over suppuration, in checking the amœboid motion of the white blood corpuscles and constringing the coats of the capillaries. Such uniformly good results, in his hands, have followed the treatment of gonorrhœa, by three or four injections daily of a solution of quinine (2½ grs. to ℥), that he has rejected all other modes. As a lotion (6 grs. to ℥), he claims to have gained very satisfactory results in the treatment of empyema; as an ointment, ten grains to the ounce of benzoated oxide of zinc, in ulcer of the leg; as a lotion, ten grains to the ounce, injected daily, in mammary abscess.

3. Dr. Libur, in a case of supposed invagination in the ileo-cæcal fossa, secured relief to the patient by injecting into the rectum a concentrated solution of bicarbonate of soda, and immediately afterwards a concentrated solution of tartaric acid.* A large quantity of gas was eliminated, which forcibly distended the intestinal tube. The gases and the fœcal matters forthwith escaped freely, and the relief was complete.

4. A young man had had an operation performed upon one of his arms, in which Esmarch's apparatus had been applied. Paralysis of motion followed, and the visiting surgeons were of the opinion that it had been caused by the pressure made upon the nerves in the application of the rubber cord. Paralysis remained for about a month. Motion was being gradually restored.

5. Saint Germain, who advised opening the trachea with a button-shaped cautery iron, has tried the following modification of this operation on a three-year-old child, who was suffering from croup. Locating the crico-thyroid membrane with his finger, he fixed the larynx firmly with one hand, while with the other he pressed a red-hot probe-pointed knife, directing the edge downward, into the larynx, just below the edge of the thyroid cartilage. There was no difficulty in doing this, and the loss of resistance showed that the knife had entered the cavity. The operator thus cut downwards through the cricoid cartilage and the first ring of the trachea. The operation was entirely bloodless. Using dilatation he

* The practice originated in this country.

readily introduced the canula. The case progressed favorably.

6. (*a.*) The third case reported (artificial anus in the right illiac region), is interesting as affording the opportunity to see outside of the abdomen, the ileo-cæcal valve and appendix vermiformis ; also as illustrating the time it took, by hydrostatic pressure, for a stream of water to travel through the rectum and colon, which did not exceed one minute. From repeated experiments by Prof. Simon, it seems to make very little difference whether the patient was in the recumbent or erect position.

(*b.*) In the fifth case reported by Dr. Bozeman (retroflexion and prolapsus of the uterus), Prof. Simon illustrated in the most practical manner the subject of rectal and vesical explorations, by the passage of the hand in the rectum. This means of diagnosis, it is said, originated with the latter.

The passage of the *hand and arm*, per anum, to a point in the abdomen that the left kidney and false ribs can be felt, and the abdominal wall, of the same side, to the median line, be lifted up at every point, upon the ends of the fingers, is a means of diagnosis which should not be overlooked.

From what he has seen and heard of this procedure, at the clinique of Prof. Simon, the reporter is convinced that the dangers and accidents supposed to attend it have been greatly exaggerated. Now and then a little incontinence of fæces is observed, which, however, continues only two or three days.

The preparation for the operation consists in complete anæsthesia and in thoroughly washing out the large bowels, by hydrostatic pressure. From two to three quarts of water usually suffice. Prof. Simon has now operated eight times through the anus for recto-vaginal fistule. Why not, he suggests, approach the male bladder in the same manner in cases of chronic catarrh and ulceration, and of great enlargement of the prostate gland, where micturition is attended with difficulty?

(*c.*) Another case served to illustrate the exploration of the female bladder, by the finger, through the urethra. Incontinence of urine had not followed the procedure. In the case under consideration, the bladder was filled with water, and the power of retention fully shown. The distention of the urethra is effected by means of a set of dilators, made of hard rubber, and ranging in size from that of the largest male catheter to that of the thumb. The dilatation is begun and carried on to the point of distention of the urethral orifice, when the latter is notched on the two sides and in front, the main seat of resistance. After this the requisite dilatation for the index finger to pass is easily reached.

Prof. Simon had turned the method to good account in a case of fungous growth from the vesical mucous membrane, which he diagnosed with the finger and detached with a gouge-shaped scraper. He had also satisfactorily employed the method in differentiating the size of a fistule, shut in by a colpocleidic operation, performed by another surgeon, the patient being dissatisfied with what she had been told was a cure. Upon examination he found the closure complete, and through the urethra only could he determine whether the fistule was small or large. Upon this point depended, he said, the justifiability or otherwise of re-establishing the vaginal tract. This method of vesical exploration with the finger showed the fistule to be small, and curable by refreshing and uniting its edges in the ordinary way. Thereupon the occluded vagina was opened and the fistule brought into view.

